# Influenza vaccine effectiveness according to frailty status in adults aged ≥ 50 years: a systematic review and meta-analysis

**DOI:** 10.1186/s12879-026-13837-y

**Published:** 2026-07-11

**Authors:** Henrique Pott, Shelly A. McNeil, Melissa K. Andrew

**Affiliations:** 1 Canadian Center for Vaccinology, Nova Scotia Health, IWK Health, Dalhousie University, Halifax, Canada; 2 Vigilare Lab, Department of Medicine, Universidade Federal de São Carlos, Rod. Washington Luis, km 235, São Carlos, SP 13656-905 Brazil; 3 Department of Medicine (Infectious Diseases), Dalhousie University, Halifax, Canada; 4 Department of Medicine (Geriatrics), Dalhousie University, Halifax, Canada

**Keywords:** Frailty, Influenza vaccines, Vaccine efficacy, Aged, Systematic review

## Abstract

**Background:**

Older adults with influenza have an elevated risk of adverse clinical outcomes. Influenza vaccine effectiveness (VE) is generally lower in this age group due to several factors. Frailty has been identified as a potential modifier of VE, but the literature on this topic is fragmented.

**Methods:**

A systematic review of studies evaluating frailty and influenza VE in adults aged ≥ 50 years. Databases were searched from inception to September 2025. The primary outcomes were laboratory-confirmed influenza (LCI), influenza-related hospitalization, and influenza-associated mortality. Random-effects models with restricted maximum likelihood estimation and Hartung-Knapp adjustment were applied when pooling was appropriate. Effect sizes were reported as odds ratios (ORs) with 95% confidence intervals (CIs).

**Results:**

Eight studies with 262,952 pooled participants were included. The prevalence of frailty ranged from 14.3% to 36.4%, with a pooled estimate of 24.3% (95% CI, 19.0%–30.4%; I²=99.8%). Four test-negative studies were pooled for LCI cases. Vaccination was associated with reduced odds of LCI (pooled OR, 0.53; 95% CI, 0.37–0.76; I²=4.0%), with a 95% prediction interval of 0.30–0.96. Stratified analyses showed pooled ORs of 0.43 (95% CI, 0.24–0.75) and 0.66 (95% CI, 0.35–1.25) in non-frail and frail individuals, respectively; no statistically significant effect modification was detected (*p* = 0.10). Hospitalization and mortality outcomes were not pooled because of heterogeneity. The certainty of evidence was low for LCI and very low for other outcomes.

**Conclusions:**

Influenza vaccination protected against LCI across frailty strata, though study heterogeneity requires cautious interpretation, and estimates were less precise for frail individuals. The elevated risk of adverse clinical outcomes in this age group supports immunization, and current data do not justify withholding vaccination.

**Supplementary Information:**

The online version contains supplementary material available at https://doi.org/10.1186/s12879-026-13837-y.

## Introduction

Seasonal influenza is a leading cause of hospitalization and death among adults aged 65 years and older, with hospitalization rates and in-hospital mortality that increase substantially with advancing age and in the presence of underlying comorbidities [1, 2]. Vaccination is the primary preventive measure; however, vaccine effectiveness (VE) declines with age, generally ranging from 30% to 50% in older adults compared to 40% to 70% in younger adults [3–5]. This reduction has been attributed to age-related immune changes (mainly immunosenescence and inflammaging) and interindividual factors such as prior infection, repeated vaccination, and comorbidities [6–8]. 

Frailty is a multidimensional construct that represents increased vulnerability to adverse outcomes [9]. It is estimated that about 10% to 25% of older adults living in the community are frail, and the prevalence is much higher among residents in long-term care facilities [10]. Literature shows that frail individuals are more likely to be hospitalized and die from influenza [11–14]. However, it remains uncertain whether frailty independently modifies influenza VE.

Current studies on the relationship between frailty and influenza VE are fragmented and vary in instruments used to measure frailty and outcomes used as correlates of VE, which limits inferences regarding effect modification [7, 8]. To our knowledge, there has been no systematic review focusing on frailty as a factor affecting influenza vaccine effectiveness.

Therefore, we systematically synthesized the available evidence to examine whether frailty modifies influenza vaccine effectiveness in adults aged 50 and older. Our focus was on clinical outcomes, including laboratory-confirmed influenza (LCI), hospitalization, and mortality. We excluded studies that only examined immunological measures of influenza VE, such as antibody levels and seroconversion rates.

## Methods

### Review protocol

This systematic review was conducted according to a pre-specified protocol registered in the PROSPERO database (CRD420251153863).

### Eligibility criteria

The eligibility criteria were specified using the Population, Exposure, Comparator, Outcomes, and Study design framework. Eligible studies enrolled adults aged ≥ 50 years in community or institutional settings who received a licensed influenza vaccine and had frailty assessed using a validated instrument, including the Frailty Index [15], Clinical Frailty Scale [9], Fried Frailty Phenotype [16], or other structured frailty measures such as the Edmonton Frail Scale [17] or the Tilburg Frailty Indicator [18]. Frailty was treated as the exposure, with its definitions applied as originally reported in each study. The comparators were individuals identified as non-frail within the same source population. Studies on relative VE that compared different vaccines formulations were also included, provided they reported results based on frailty levels.

The primary outcomes of interest were LCI infection, hospitalization, and mortality. The secondary outcomes included symptomatic disease.

Eligible designs included randomized controlled trials, cohort studies, and test-negative case-control studies, including those using administrative healthcare databases. Cross-sectional, ecological, modeling-only studies, case reports, and conference abstracts without complete data were excluded. Only studies published in English, French, Portuguese or Spanish were considered, without restrictions on the publication status or year.

### Information sources and search strategy

Electronic database searches were conducted in MEDLINE (via PubMed), Embase, and Scopus up to September 2025, with no restriction on publication date. The reference lists of the included studies and relevant reviews were screened to identify additional records.

The search strategy was developed in collaboration with a medical librarian. Controlled vocabulary terms were combined with free-text keywords related to older adults, frailty, and influenza vaccinations. Synonyms and truncation were used to enhance the sensitivity. The Boolean operators AND and OR structured the search logic across three conceptual domains: population, frailty, and vaccination. No restrictive methodological filters were applied. The full search strategies for each database are provided in the Supplementary Material to ensure reproducibility.

### Study selection

Records were imported into Rayyan tool plataform for duplicate removal and screening. Titles and abstracts were independently screened by two blinded reviewers. Full-text articles of potentially eligible studies were independently assessed by the same reviewers. Disagreements at any stage were resolved through discussion, and persistent discrepancies were adjudicated by a third reviewer, if necessary. The reasons for exclusion at the full-text stage were documented. The selection process is illustrated in a PRISMA flow diagram.

### Data extraction

Data were independently extracted using a standardized pilot-tested extraction form. The extracted variables included study characteristics, participant demographics, frailty instruments and categorization thresholds, vaccination details, statistical methods, effect estimates with corresponding measures of precision, funding sources, and reported conflicts of interest.

When both crude and adjusted estimates were reported, the adjusted effect estimates were preferentially extracted. When multiple adjusted models were presented, estimates controlling for the most comprehensive set of confounders were selected.

### Risk of bias and certainty of evidence

The risk of bias was assessed according to the study design. Randomized trials were evaluated using the Risk of Bias (RoB) 2 tool [19]. Non-randomized studies were assessed using the ROBINS-I tool [20]. All assessments were performed independently by two reviewers. Disagreements were resolved through consensus discussions. Overall risk of bias judgments were derived from domain-level assessments according to the tool-specific guidance.

The certainty of evidence for each outcome was evaluated using the Grading of Recommendations, Assessment, Development and Evaluations (GRADE) framework [21]. For randomized controlled trials, five downgrading factors were considered: risk of bias, inconsistency, indirectness, imprecision, and publication bias. For observational studies, in addition to these downgrading factors, three upgrading factors were also evaluated: large magnitude of effect, dose-response gradient, and the effect of plausible residual confounding. Each factor judged as serious resulted in one level downgrade; factors judged as very serious resulted in two levels downgrade. Evidence was classified into four certainty categories: high, moderate, low, and very low. These evaluations informed the interpretation but did not modify the pre-specified analytic strategy.

### Statistical analysis and data synthesis

Frailty prevalence was pooled using a random-effects generalized linear mixed model with a logit link, with the between-study variance estimated using the restricted maximum likelihood. Pooled estimates are presented with 95% confidence intervals (CIs). Heterogeneity was quantified using Cochran’s Q, I^2^, and τ^2^ tests.

For LCI infection derived from test-negative case-control studies, effect estimates were synthesized when comparable measures were available. When VE was reported as a percentage rather than an odds ratio, effect measures were back-transformed to the odds ratio (OR) prior to pooling using the relationship OR = 1 − (VE/100). Given the heterogeneity in frailty instruments, categories were dichotomized as frail versus non-frail or lowest frailty level. Pooled ORs were calculated using inverse-variance weighting under a random-effects model with restricted maximum likelihood estimation. The Hartung-Knapp adjustment was applied to account for uncertainty in variance estimation. Effect modification by frailty status was examined through stratified analyses and, when applicable, formal tests for subgroup differences using Cochran’s Q, with statistical significance defined as *p* < 0.05. Prediction intervals were calculated to reflect the dispersion of true effects across settings.

Hospitalization and mortality outcomes were not pooled because of heterogeneity in the design, vaccine formulation, frailty definition, and effect measures. These outcomes were synthesized narratively.

All analyses were conducted using R version 4.5.3 (R Foundation for Statistical Computing, Vienna, Austria) with RStudio 2026.04.0 + 526 “Globemaster Allium” Release.

## Results

### Study identification and selection

**Fig. 1:**
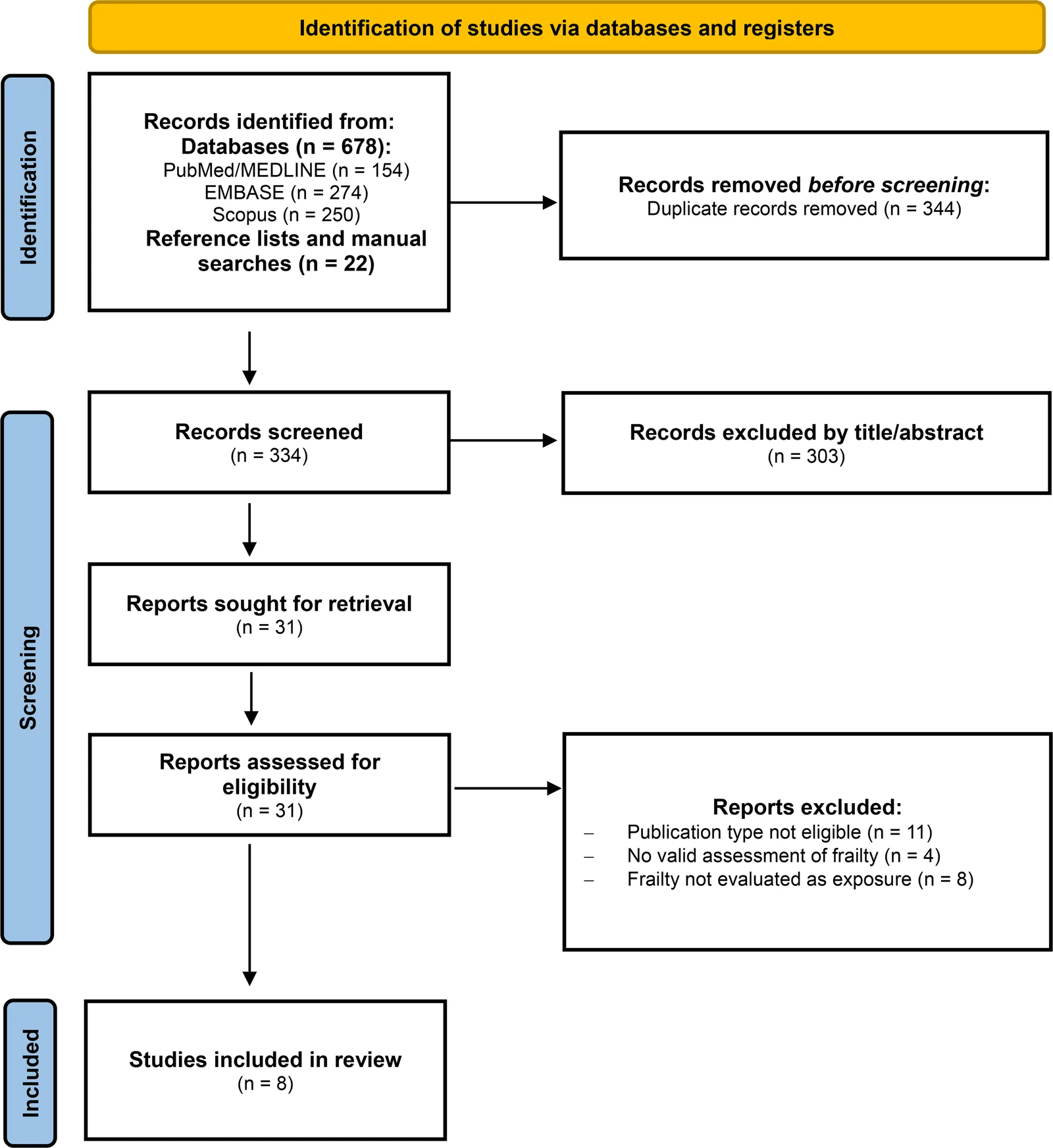
PRISMA-based flowchart of the systematic review and study selection

The initial systematic search across electronic databases identified 678 citations, supplemented by 22 records retrieved through manual reference chain tracking. Following the removal of duplicates, 334 unique titles and abstracts were screened, resulting in the exclusion of 303 records that did not meet the preliminary eligibility criteria. Of the 31 articles retained for full-text evaluation, 23 were subsequently excluded (primarily due to ineligible publication formats, lack of validated frailty assessments, or failure to isolate frailty as an exposure of interest). Ultimately, eight studies met all the inclusion criteria, comprising a pooled cohort of 262,952 participants (Fig. 1).

### Study characteristics and population profile

The evidence base integrated four test-negative case-control studies, two randomized clinical trials, and two cohort investigations, spanning the influenza seasons from 2004 to 2005 to 2021–2022. (Table 1) Most studies were conducted in North America, with additional data from Italy and Denmark. Participant demographics reflected a focus on aging populations; several investigations were restricted to adults aged ≥ 65 years (including a long-term care cohort with a mean age of 84 years), whereas two test-negative analyses evaluated broader adult cohorts with mean ages typically ranging from the late 50s.

**Table 1:** Characteristics of the included studies

Study	Country	Design	Seasons	Population (*N*)	Age	Frailty Measure	Vaccine Exposure	Outcomes Reported
Yao et al. (2011)	USA	Prospective cohort	2004–2005	1,227	Mean 84.5	Fried Frailty Phenotype	TIV	ILI; LCI; Hosp; Mort
Diaz Granados et al. (2015)	USA, Canada	Randomized clinical trial	2011–2012	31,989	Mean 73	Frailty-associated condition count	HD-IIV vs. SD-IIV	LCI; Reactogenicity; Immunogenicity
Petrie et al. (2016)	USA	Test-negative case-control	2014–2015	624	≥ 65 years (35.3%)	5-item Frailty Score	TIV/QIV (mixed)	LCI; VE
Talbot et al. (2016)	USA	Test-negative case-control	2006–2007, 2007–2008, 2008–2009, 2010–2011, 2011–2012	1023	Median 60.5	Frailty Index	LAIV, HD-IIV, SD-IIV	LCI; VE
Andrew et al. (2017)	Canada	Test-negative case-control	2011–2012	884	≥ 65 years (100%)	Frailty Index	TIV	LCI; VE; Hosp; Mort
Fausto et al. (2018)	Italy	Retrospective population-based cohort study	2014–2015	213,261	≥ 65 years (100%)	Frailty Index	NA	Mort
Ferdinands et al. (2019)	USA	Test-negative case-control	2013–2014, 2014–2015, 2015–2016	1,467	Mean 58	5-item Frailty Score	NA	LCI; VE; Hosp
Espersen et al. (2025)	Denmark	Randomized pragmatic trial	2021–2022	12,477	Mean 71.7	Hospital Frailty Risk Score	HD-IIV vs. SD-IIV	LCI; Hosp; Mort; All-cause Hosp

Notes: Population (N) refers to the total number of enrolled participants in each study

Age is reported as mean, median, or percentage of participants above a defined threshold, as available in each source study. NA = vaccine formulation not reported; study focused on vaccination status rather than specific formulation

Andrew et al. (2017) evaluated standard trivalent inactivated vaccines only

Seasons refer to the influenza season(s) under analysis; test-negative studies spanned multiple seasons, whereas trials and the cohort study evaluated a single season

Abbreviations: HD-IIV = high-dose inactivated influenza vaccine; SD-IIV = standard-dose inactivated influenza vaccine; TIV = trivalent inactivated vaccine; QIV = quadrivalent inactivated vaccine; LAIV = live attenuated influenza vaccine; ILI = influenza-like illness; LCI = laboratory-confirmed influenza; Hosp = influenza-associated hospitalization; Mort = influenza-associated mortality; VE = vaccine effectiveness

The methodological approaches to frailty ascertainment were heterogeneous. Deficit-accumulation indices were employed in three studies, the Fried phenotype in one study, and abbreviated frailty scores in two studies. One randomized trial utilized a clinical condition-based classification, whereas another leveraged the administrative data-derived Hospital Frailty Risk Score. Vaccine exposures remained consistent with contemporaneous public health policies, ranging from standard-dose trivalent or quadrivalent formulations in observational contexts to head-to-head comparisons of high-dose (HD) versus standard-dose (SD) vaccines in trial settings. Outcomes focused primarily on laboratory-confirmed influenza, although definitions extended to influenza-like illness and secondary endpoints, including influenza-associated or all-cause hospitalization and mortality.

**Fig. 2:**
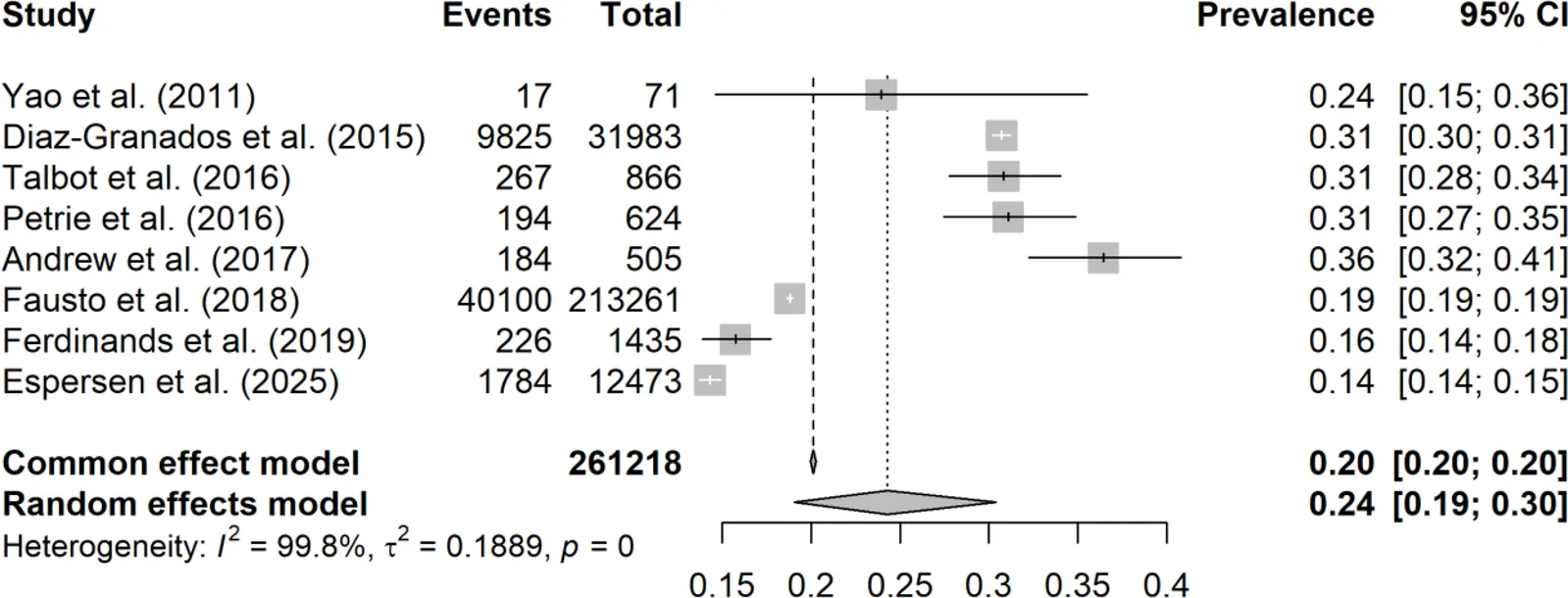
Random-effects meta-analysis of frailty prevalence in the included studies

Across these diverse clinical environments, frailty prevalence ranged from 14.3% to 36.4%. The meta-analysis using a random-intercept logistic regression model yielded a pooled prevalence of 24.3% (95% CI, 19.0%-30.4%). Between-study heterogeneity was substantial (τ² = 0.189, I² = 99.8%, Cochran’s Q = 2856.07, *p* < 0.001). The common-effect estimate was lower at 20.1% (95% CI, 20.0%-20.3%); however, given the magnitude of heterogeneity, the inference was based on the random-effects model. (Fig. 2)

### Methodological quality and risk of bias assessment

The internal validity of the included evidence reflected the divergent risk profiles inherent to the study architecture. (Table 2) Systematic assessment using the RoB 2 tool for randomized trials [13, 22] indicated a spectrum from low risk to moderate concern; the latter was primarily driven by incomplete participant blinding and a reliance on registry-based outcome ascertainment [13]. In contrast, observational studies evaluated using the ROBINS-I framework exhibited moderate to serious risk of bias.

**Table 2:** Risk of bias summary

Study	Design	Domain						Overall RoB	Impact on synthesis
		1	2	3	4	5	6/7		
Yao et al. (2011)	PC	✖	●	●	—	●	✖	Serious	Descriptive outcomes only; not used for VE synthesis
Diaz Granados et al. (2015)	RCT	✔	✔	✔	✔	✔	—	Low	Contributes comparative efficacy estimates; analyzed separately from TNDs
Petrie et al. (2016)	TND	●	✔	●	✔	●	●	Moderate	Contributes adjusted VE estimates; interpreted cautiously
Talbot et al. (2016)	TND	●	✔	●	✔	●	●	Moderate	Included in narrative pattern assessment
Andrew et al. (2017)	TND	✖	●	✖	●	●	●	Serious	Narrative direction only; excluded from sensitivity emphasis
Fausto et al. (2018)	RC	✖	●	✔	—	●	●	Serious	Included narratively; high risk of residual confounding
Ferdinands et al. (2019)	TND	●	✔	●	✔	●	●	Moderate	Supports consistency of direction; not pooled
Espersen et al. (2025)	RCT	●	✔	●	●	✔	—	Some concerns	Provides hazard ratio estimates; design heterogeneity precludes pooling

Notes: ✔ Low risk; ● some concerns/moderate risk; ✖ serious/high risk; — = not applicable

RoB 2 domains (applied to randomized trials): 1 = randomization process; 2 = deviations from intended interventions; 3 = missing outcome data; 4 = outcome measurement; 5 = selection of reported results. ROBINS-I domains (applied to observational studies): 1 = confounding; 2 = selection of participants; 3 = classification of interventions; 4 = deviations from intended interventions; 5 = missing data; 6 = measurement of outcomes; 7 = selection of reported results. Overall risk of bias is reported using tool-specific terminology: low, some concerns, or high risk for RoB 2; and low, moderate, serious, or critical for ROBINS-I

Across observational designs, confounding, participant selection, and imprecision within frail strata were the most frequently contributing domains to elevated risk

Abbreviations: PC = Prospective cohort; RC = Retrospective cohort; RCT = Randomized Clinical Trial; TND = Test-negative case-control design

Residual confounding remained the predominant threat to validity, particularly regarding frailty-associated health status and prior healthcare use. While test-negative designs effectively mitigate differential healthcare-seeking behavior, they remain susceptible to confounding by indication (”unhealthy vacinee”) and/or the ”healthy vaccinee” effect, depending on the context. Notably, three studies [11, 12, 23] were classified as having a serious risk of bias, largely due to insufficient adjustment for key covariates or small sample sizes. These methodological constraints were explicitly integrated into the GRADE-based assessment of evidence certainty, which moderated the strength of subsequent clinical inferences.

### Vaccine effectiveness against laboratory-confirmed influenza

Laboratory-confirmed influenza was evaluated in seven studies. Randomized trial data provide the strongest evidence base for this outcome. A large multicenter trial comparing HD and SD inactivated vaccines reported a superior reduction in PCR-confirmed influenza with the HD formulation across all age groups and among those with frailty-associated comorbidities [22]. Similarly, a pragmatic Danish trial found that HD vaccination was associated with a lower hazard of hospitalization for pneumonia or influenza, regardless of frailty status, with no statistical evidence of an interaction between vaccine type and frailty level [13]. 

**Fig. 3:**
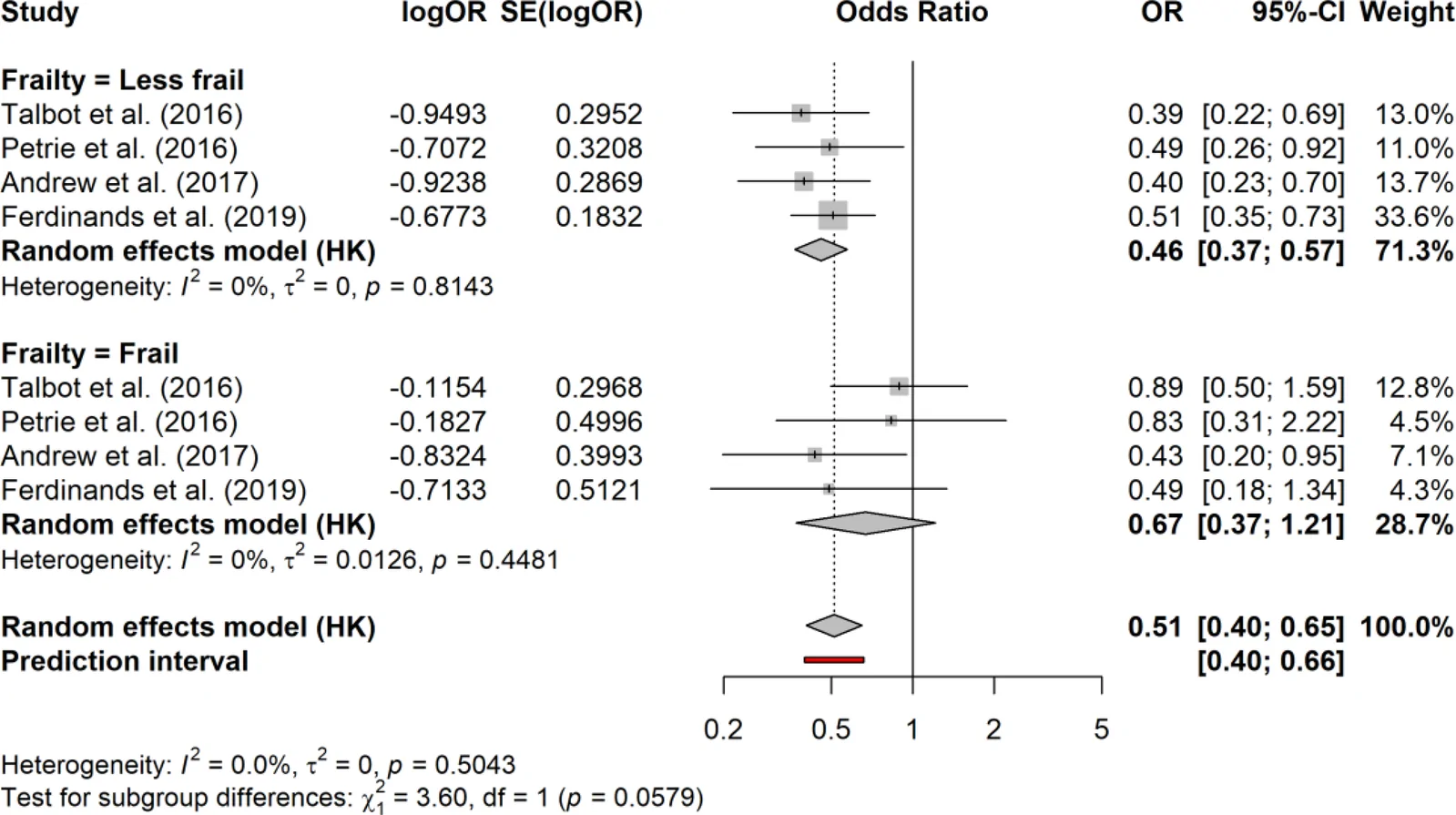
Pooled odds ratios for laboratory-confirmed influenza according to frailty status

These findings are further supported by convergent observational evidence. The quantitative synthesis integrated four test-negative case-control studies [12, 24–26]. To ensure comparability across heterogeneous frailty instruments, data were dichotomized into non-frail and frail categories; intermediate and extreme classifications (e.g., “pre-frail” and “most frail”) were excluded to prevent unit-of-analysis errors within the multilevel scales. Eight distinct strata were pooled using inverse-variance weighting with restricted maximum likelihood estimation and Hartung-Knapp adjustments. Vaccination was associated with lower odds of laboratory-confirmed influenza, yielding a pooled OR of 0.53 (95% CI, 0.37–0.76; *p* = 0.004). Statistical heterogeneity was negligible (τ² = 0.035; I² = 4.0%; Cochran’s Q = 7.30; *p* = 0.40), and the 95% prediction interval (0.30–0.96) suggested consistent effects across future studies. (Fig. 3)

Stratified analysis demonstrated a pooled OR of 0.43 (95% CI, 0.24–0.75) in non-frail participants compared to 0.66 (95% CI, 0.35–1.25) in the frail subgroup. While effect estimates were directionally consistent across both groups, confidence intervals were wider in frail populations. Formal testing for subgroup differences did not reveal statistical evidence of effect modification by frailty status (Cochran’s Q = 2.70; *p* = 0.10). (Fig. 3)

In a prospective cohort of community-dwelling older adults, serologically confirmed infection was identified in 15.5% of participants; notably, incidence rates followed a significant frailty gradient, rising from 5% among non-frail individuals to 29% in the frailest stratum (*p* = 0.02) [11]. Despite this trend, event counts remain limited, necessitating cautious interpretation of point estimates.

### Influenza-associated hospitalization, mortality, and symptomatic disease

Hospitalization and mortality outcomes were not pooled because of heterogeneity in design, comparator structure, vaccine formulation, frailty definition, and effect measures, and were therefore synthesized narratively.

#### Hospitalization

Randomized evidence on influenza-associated hospitalization is provided by the pragmatic Danish trial, in which reduced hospitalization rates for pneumonia or influenza were observed in both low and intermediate/high frailty groups with HD vaccination. Formal interaction testing revealed no evidence of a differential vaccine effect based on frailty status [13]. Across test-negative investigations, adjusted vaccine effectiveness estimates against influenza-associated hospitalization generally ranged between 43% and 58%, with largely overlapping confidence intervals across frailty strata, providing no consistent evidence of differential protection by frailty status [12, 24, 25]. 

#### Mortality

In a large multicenter randomized dose-comparison trial, a statistical interaction between frailty and vaccine formulation was identified for all-cause mortality (*p* = 0.02), with HD vaccination associated with improved survival among participants with lower frailty [13]. However, small event counts and multiple stratified comparisons raise the possibility of a chance finding, and confidence in the observed interaction is limited. In a population-based cohort, the absence of vaccination was associated with a higher risk of all-cause mortality (adjusted RR 1.36; 95% CI, 1.27–1.45) and pneumonia/influenza-related death (RR 1.43; 95% CI, 1.02-2.00) after adjusting for frailty index, with this elevated risk persisting across all frailty strata [23]. 

#### Symptomatic disease

One cohort reported burden of influenza-like illness (ILI), observing a steep clinical gradient in incidence with increasing frailty [11]. ILI rates rose from 9% in non-frail individuals to 53% among those in the highest frailty category (*p* = 0.002), demonstrating a marked gradient across frailty categories.

### Certainty of evidence

The certainty of the evidence was limited across all outcomes. (Table 3) For laboratory-confirmed influenza, the certainty was rated as low. All pooled estimates were derived from observational test-negative studies, which exhibited heterogeneity in covariate adjustment, with at least one analysis lacking such adjustment. Residual confounding and partial reliance on self-reported vaccination status were identified as potentially serious limitations. Although statistical heterogeneity was low and the direction of effect was consistent, precision was limited, especially in frail strata, where confidence intervals were wide and often crossed the null. The 95% prediction interval (0.30–0.96) included values close to unity, and the overall information size was relatively small. The certainty of the evidence for influenza-related hospitalizations was also judged to be very low. Frailty-stratified analyses were limited, event counts were small, and observational estimates were subject to moderate-to-serious risk of bias. Mortality outcomes were supported by sparse and heterogeneous data, with imprecise estimates and inconsistent interaction testing between vaccination and frailty, resulting in very-low certainty. The secondary outcomes were constrained by inconsistent definitions and incomplete reporting. Publication bias was not formally assessed because of the small number of studies contributing to the pooled analyses. Overall, confidence in the effect estimates across frailty strata was limited.

**Table 3:** Summary of findings: Influenza vaccine effectiveness according to frailty status

Outcome	Population	No. of Studies (Strata)	Study Design	Risk of Bias	Inconsistency	Indirectness	Imprecision	GRADE Certainty	Key Estimate
Laboratory-confirmed influenza (HD vs. SD; overall)	Adults ≥ 50 years, frail and non-frail	2 studies	Randomized controlled trial	Low-moderate	Not serious	Not serious	Moderate	⊕⊕⊕○ Moderate	HD superior to SD across all age groups and frailty-associated comorbidities [22]; no interaction by frailty status [13]
Laboratory-confirmed influenza (overall)	Adults ≥ 50 years, frail and non-frail	4 studies (8 strata)	Test-negative case-control	Moderate-serious	Not serious (I²=4.0%)	Serious¹	Serious²	⊕⊕○○ Low	OR 0.53 (95% CI, 0.37–0.76); PI 0.30–0.96
Laboratory-confirmed influenza (non-frail)	Adults ≥ 50 years, non-frail	4 studies	Test-negative case-control	Moderate-serious	Not serious	Serious¹	Serious²	⊕⊕○○ Low	OR 0.43 (95% CI, 0.24–0.75)
Laboratory-confirmed influenza (frail)	Adults ≥ 50 years, frail	4 studies	Test-negative case-control	Moderate-serious	Not serious	Serious¹	Very serious³	⊕○○○ Very low	OR 0.66 (95% CI, 0.35–1.25)
Influenza-associated hospitalization (HD vs. SD)	Adults ≥ 65 years, frail and non-frail	1 study	Randomized controlled trial	Low-moderate	Not serious	Not serious	Serious²	⊕⊕○○ Low	Reduced hospitalization in both low and intermediate/high frailty groups; no interaction by frailty status [13]
Influenza-associated hospitalization (observational)	Adults ≥ 65 years, frail and non-frail	3 studies	Test-negative case-control	Moderate-serious	Serious⁴	Serious¹	Serious²	⊕○○○ Very low	VE 43%-58% (overlapping CIs across frailty strata)
All-cause mortality	Adults ≥ 65 years, frail and non-frail	2 studies	Cohort + RCT	Serious	Very serious⁵	Serious¹	Very serious³	⊕○○○ Very low	RR 1.36 unvaccinated (95% CI, 1.27–1.45) [23]; interaction *p*=0.02 (RCT, frailty × formulation) [13]
Influenza-like illness	Adults ≥ 50 years, frail and non-frail	1 study	Prospective cohort	Serious	Not assessable	Serious¹	Very serious³	⊕○○○ Very low	ILI rate 9% (non-frail) to 53% (frail); *p*=0.002

Notes:

¹ Indirectness: heterogeneity in frailty instruments and thresholds across studies limits direct comparability

² Serious imprecision: confidence intervals wide, particularly in frailty-stratified analyses; overall information size relatively small

³ Very serious imprecision: confidence intervals cross the null or are based on single studies with small event counts

⁴ Serious inconsistency: heterogeneity in study design, comparator structure, and frailty definitions precluded pooling

⁵ Very serious inconsistency: discordant findings between observational and randomized evidence; inconsistent interaction testing

## Discussion

Influenza vaccination in older adults is generally evaluated as a uniform preventive strategy for infectious diseases [27]. Our synthesis suggests that while protection against LCI infection remains evident across the aging continuum, the precision of effect estimates varies across the frailty spectrum. These findings highlight a clinically important uncertainty: individuals at the highest baseline risk for severe outcomes are also those in whom vaccine effectiveness estimates are least precise.

Randomized trial data yielded the strongest available evidence on influenza VE in the context of frailty. A multicenter study comparing HD and SD inactivated vaccines found that the HD formulation had a higher VE across all age groups, both for participants without high-risk comorbidities and those with at least one, as well as for individuals with a condition associated with frailty [22]. A Danish trial similarly found that HD vaccination was associated with a lower hazard of hospitalization for pneumonia or influenza regardless of frailty status, with no statistical evidence of an interaction between vaccine type and frailty level [13]. Overall, these studies demonstrate that influenza vaccines, especially those with enhanced formulations, offer significant protection for older adults regardless of their level of frailty, with no indication that frailty diminishes the vaccine’s effectiveness.

These randomized findings are further supported by observational evidence. Across diverse healthcare settings, a protective association was observed in both frail and non-frail adults, though confidence intervals were wider among frail individuals [11–13, 22–26]. The quantitative synthesis of four test-negative case-control studies yielded a pooled OR of 0.53 (95% CI, 0.37–0.76), with negligible statistical heterogeneity (I²=4.0%) and a 95% prediction interval of 0.30–0.96, suggesting consistent effects across future study contexts. Stratified analyses demonstrated a pooled OR of 0.43 (95% CI, 0.24–0.75) among non-frail participants and 0.66 (95% CI, 0.35–1.25) among frail individuals. Although effect estimates were directionally consistent across both groups, confidence intervals were wider in frail populations, and formal testing did not reveal statistical evidence of effect modification by frailty status (*p* = 0.10). These findings highlight a clinically important uncertainty: individuals at the highest baseline risk for severe outcomes are also those in whom vaccine effectiveness estimates are least precise. LCI emerges as the outcome with the greatest evidentiary support, and the consistency of the protective signal across frailty strata (despite imprecision) reinforces the rationale for universal vaccination in this population.

Data on enhanced formulations, including HD and adjuvanted vaccines, consistently demonstrate superior protection compared with SD formulations. However, evidence directly comparing the relative effectiveness of enhanced products across frailty strata remains limited [13, 14, 22, 28]. Notably, prior work from our group demonstrated that, after accounting for frailty, the adjuvanted trivalent inactivated influenza vaccine provided significantly greater protection against influenza-related hospitalizations in older adults than non-adjuvanted formulations [14]. This finding aligns with current public health recommendations favoring the use of HD or adjuvanted formulations for adults aged ≥ 65 years, a recommendation that becomes even more compelling when considering frail individuals, who face a substantially higher burden of influenza-related morbidity. Immunologic findings, however, are less consistent: some settings report attenuated hemagglutination inhibition responses with increasing frailty [11], while a study comparing HD and SD inactivated vaccines found higher early post-vaccination titers among frail participants, particularly in the HD group [29]. Together, these observations suggest that the immunologic expression of frailty may depend on the vaccine formulation and antigen dose as well as on host factors and that persistently elevated clinical risk among frail individuals likely reflects factors beyond peak antibody titers, including frailty-associated dysreguation of immune responses and residual confounding from the “(un)healthy vaccinee” effect.

Frailty is consistently identified as an important predictor of adverse health outcomes across a multitude of fields, including outcomes after respiratory infections and illnesses [30]. Even so, the evidence in relation to influenza vaccine effectiveness across stratified grades of frailty remains limited and has not been examined in a unified manner, and several methodological barriers hinder interpretation. (Table 3) Dichotomizing frailty for meta-analyses inevitably biases the vulnerability gradient. Although formal testing did not demonstrate statistically significant interaction by frailty status, the limited number of events within higher-frailty strata restricts the ability to exclude clinically meaningful differences. Underlying this uncertainty is plausible immunobiological rationale for why frailty might constrain vaccine-induced protection independently of chronological age. The convergence of immunosenescence, chronic low-grade inflammation (often termed “inflammaging”) and diminished physiological reserve may collectively impair both the generation and durability of vaccine-induced immunity [28, 31–33]. These mechanisms are not simply age-dependent; they reflect a trajectory of cumulative biological decline that chronological age alone fails to capture.

The diversity of frailty instruments used across the included studies further exacerbates this challenge. Various methods, such as deficit-accumulation indices, phenotypic classifications, abbreviated clinical scores, and administrative risk scores, were employed, yet these measures have not been consistently calibrated against one another within this body of literature. This is important because frailty, in essence, describes the divergence in health trajectories that accumulates over a lifetime, where frail individuals of the same chronological age exhibit far more variation in their biological profiles than their non-frail counterparts [34]. When this heterogeneity is captured differently across studies, pooled estimates end up conflating distinct populations, which further limits the precision of subgroup inferences and, consequently, the interpretation of differential effectiveness.

To our knowledge, no prior systematic review has formally examined frailty as a modifier of influenza vaccine effectiveness through pooled analysis across multiple study designs. Earlier investigations have relied predominantly on single-cohort estimates or aggregate effectiveness data, typically lacking both the granular frailty characterization and the statistical power required to evaluate whether biological vulnerability alters the magnitude of vaccine-conferred protection [12, 14]. By synthesizing evidence across studies, the present analysis extends beyond conventional age-based risk stratification and raises the question of whether frailty assessment could serve as a more precise basis for targeting preventive strategies in older adults [27]. 

From a practical standpoint, our results argue against the use of chronological age as a sufficient proxy for risk stratification [33]. Frailty assessment may inform decisions regarding vaccine formulation and the deployment of complementary preventive measures (for example, in long-term care settings) [35]. What remains uncertain is the magnitude of the incremental benefit and the optimal strategy to achieve it.

Several limitations merit acknowledgment. The evidence base was restricted to eight studies, most of which were conducted in North America, with only limited representation from European populations. In addition, the restriction to English, French, Portuguese, and Spanish-language publications may have resulted in underrepresentation of data from other geographic regions. These factors may limit the external validity of the findings, particularly given potential differences across healthcare systems, influenza surveillance practices, vaccination coverage, diagnostic ascertainment, and baseline population characteristics in older adults. Accordingly, the present findings are likely most applicable to high-income settings with established surveillance infrastructure, and extrapolation to other epidemiological contexts should be undertaken cautiously.

Taken together, this systematic review and meta-analysis provides evidence that influenza vaccination confers protection against LCI infection across frailty strata, although the substantial heterogeneity across included studies warrants cautious interpretation of the pooled estimates. Effect estimates are less precise and statistically more uncertain among frail individuals, and frailty-stratified evidence for severe clinical outcomes remains insufficient to support definitive conclusions. The elevated baseline risk inherent to this population sustains the rationale for immunization prioritization, and the available data do not support withholding vaccination on the basis of frailty status. Advancing this field will require study designs that treat frailty as a pre-specified structural determinant, harmonized outcome frameworks, and analytic approaches capable of resolving the incremental benefit of optimized vaccine strategies across the full frailty continuum.

## Supplementary Information

Below is the link to the electronic supplementary material.

Supplementary Material 1

## Data Availability

The datasets examined in this study originated from articles that have been previously published and referenced in this paper. The dataset compiled for the meta-analysis can be obtained from the corresponding author upon reasonable requests.
