# Supplementary material for: Influenza vaccine effectiveness according to frailty status in adults aged ≥ 50 years: a systematic review and meta-analysis
Source: BMC Infect Dis. 2026 Jul 11;26:1778. doi: 10.1186/s12879-026-13837-y (PMC13640225; doi:10.1186/s12879-026-13837-y)
Supplement: Supplementary file 1 [file 12879_2026_13837_MOESM1_ESM.docx]

# **Search Strategy**

## **PubMed**

(

"aged"[MeSH Terms]

OR "middle aged"[MeSH Terms]

OR "older adults"[Title/Abstract]

OR elderly[Title/Abstract]

OR "50 years and older"[Title/Abstract]

)

AND

(

"frailty index"[Title/Abstract]

OR "clinical frailty scale"[Title/Abstract]

OR "fried frailty phenotype"[Title/Abstract]

OR frail*[Title/Abstract]

OR "frailty syndrome"[Title/Abstract]

OR "frailty score"[Title/Abstract]

OR "frailty measure"[Title/Abstract]

OR frailty[MeSH Terms]

)

AND

(

"influenza vaccines"[MeSH Terms]

OR "influenza vaccine"[Title/Abstract]

OR "live attenuated influenza vaccine"[Title/Abstract]

OR "inactivated influenza vaccine"[Title/Abstract]

OR "trivalent influenza vaccine"[Title/Abstract]

OR "quadrivalent influenza vaccine"[Title/Abstract]

OR "flu vaccine"[Title/Abstract]

OR LAIV[Title/Abstract]

OR IIV[Title/Abstract]

)

## **Embase**

(

'aged'/exp OR 'middle aged'/exp OR 'older adults':ti,ab OR elderly:ti,ab OR '50 years and older':ti,ab

)

AND

(

'frailty index':ti,ab OR 'clinical frailty scale':ti,ab OR 'fried frailty phenotype':ti,ab OR frail*:ti,ab OR 'frailty syndrome':ti,ab OR 'frailty score':ti,ab OR 'frailty measure':ti,ab OR 'frailty'/exp

)

AND

(

'influenza vaccine'/exp OR 'influenza vaccine':ti,ab OR 'live attenuated influenza vaccine':ti,ab OR 'inactivated influenza vaccine':ti,ab OR 'trivalent influenza vaccine':ti,ab OR 'quadrivalent influenza vaccine':ti,ab OR 'flu vaccine':ti,ab OR LAIV:ti,ab OR IIV:ti,ab

)

## **Scopus**

( TITLE-ABS-KEY ( "aged" ) OR TITLE-ABS-KEY ( "middle aged" ) OR TITLE-ABS-KEY ( "older adults" ) OR TITLE-ABS-KEY ( elderly ) OR TITLE-ABS-KEY ( "50 years and older" ) AND TITLE-ABS-KEY ( "frailty index" ) OR TITLE-ABS-KEY ( "clinical frailty scale" ) OR TITLE-ABS-KEY ( "fried frailty phenotype" ) OR TITLE-ABS-KEY ( frail* ) OR TITLE-ABS-KEY ( "frailty syndrome" ) OR TITLE-ABS-KEY ( "frailty score" ) OR TITLE-ABS-KEY ( "frailty measure" ) OR TITLE-ABS-KEY ( frailty ) AND TITLE-ABS-KEY ( "influenza vaccine" ) OR TITLE-ABS-KEY ( "live attenuated influenza vaccine" ) OR TITLE-ABS-KEY ( "inactivated influenza vaccine" ) OR TITLE-ABS-KEY ( "trivalent influenza vaccine" ) OR TITLE-ABS-KEY ( "quadrivalent influenza vaccine" ) OR TITLE-ABS-KEY ( "flu vaccine" ) OR TITLE-ABS-KEY ( LAIV ) OR TITLE-ABS-KEY ( IIV ) )
